# Supplementary material for: Epidemiological Investigation of Hospital Transmission of Corynebacterium striatum Infection by Core Genome Multilocus Sequence Typing Approach
Source: Microbiol Spectr. 2022 Dec 20;11(1):e01490-22. doi: 10.1128/spectrum.01490-22 (PMC9927548; doi:10.1128/spectrum.01490-22)
Supplement: Supplemental file 1 — Supplemental material. Download spectrum.01490-22-s0001.pdf, PDF file, 1.4 MB [file spectrum.01490-22-s0001.pdf]

Figure S1. The numbers of loci and genomes in every exclusion threshold level for the 271 genomes that created the cgMLST scheme. The numbers of loci found in 100%, 99.5%, 99%, and 95% of genomes are shown. The straight line represents the threshold selected for further analysis, which defines the maximum loss of loci by genomes.

Figure S2. The numbers of loci and genomes in every exclusion threshold level for the 31 validation genomes and 233 genomes that created the cgMLST scheme. The numbers of loci found in 100%, 99.5%, 99%, and 95% of genomes are shown. The straight line represents the threshold selected for further analysis, which defines the maximum loss of loci by genomes.

Figure S3. Neighbour-joining tree of the 263 *C. striatum* genomes as determined on the basis of cgMLST allelic profiles, showing the association between CGs and STs.

Figure S4. Topological correspondence between the cgMLST neighbor joining tree (right) and the SNP-based maximum-likelihood tree (left) is shown as a tanglegram.

Figure S5. Minimum-spanning tree based on cgMLST allelic profiles of 30 validation *C. striatum* isolates. Nodes corresponding to unique allelic profiles are coloured according to their corresponding isolate sources (a), departments (b) and floors (c).

Figure S6. Plot showing the Dunn index for clustering thresholds, ranging from 1 to 40 allelic differences.

Table S1 List of 271 *C. striatum* genomes obtained from the NCBI genome database on August 29, 2021.

Table S2 Primer sequences for *gyrA*, *gyrB*, *hsp65*, *rpoB*, *secA1*, and *sodA*.

Table S3 The CG distribution of 263 *C. striatum* strains.

Table S4 MLST locus polymorphism signature.

Table S5 The ST distribution of 263 *C. striatum* strains.

Table S6 validation strains and their characteristics.

Table S7 Hospital transmission investigation results of 213 published nosocomial transmission strains isolated from three hospitals in three regions of China.

Table S8 The antibiotic resistance phenotype of 213 *C. striatum* strains.

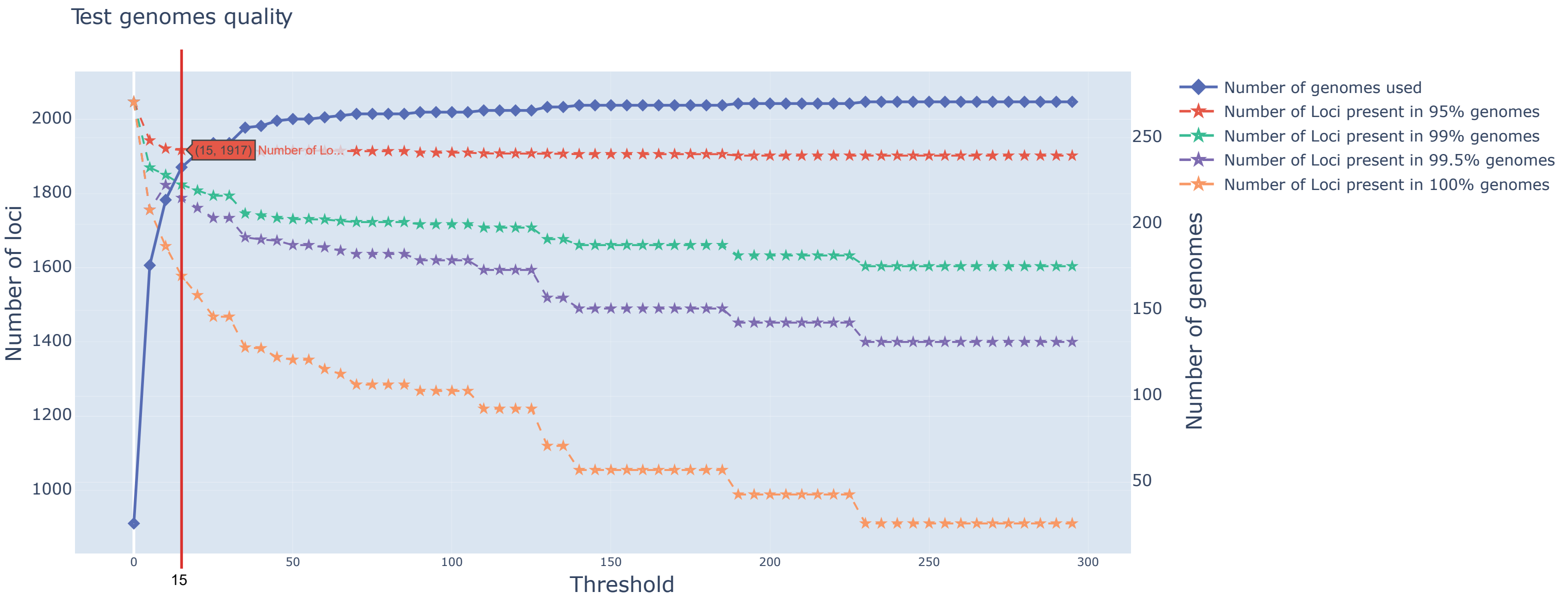
Test genomes quality
Number of genomes used
Number of Loci present in 95% genomes
Number of Loci present in 99% genomes
Number of Loci present in 99.5% genomes
Number of Loci present in 100% genomes
Number of loci
Number of genomes
Threshold
(15, 1917)
15

Figure S1

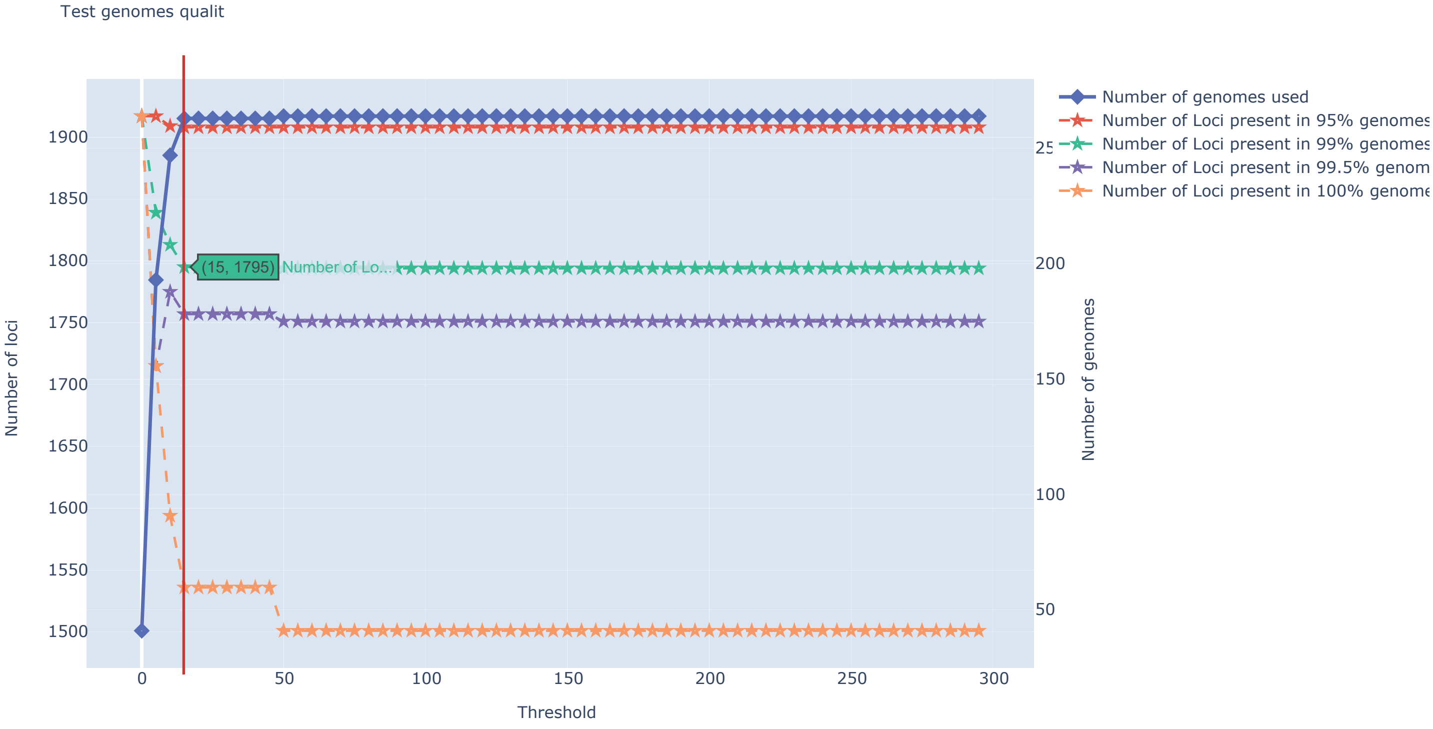
Test genomes qualit
Number of genomes used
Number of Loci present in 95% genomes
Number of Loci present in 99% genomes
Number of Loci present in 99.5% genom
Number of Loci present in 100% genome
(15, 1795)
Number of Lo...
Number of loci
Number of genomes
Threshold
1900
1850
1800
1750
1700
1650
1600
1550
1500
200
150
100
50
0
50
100
150
200
250
300

Figure S2

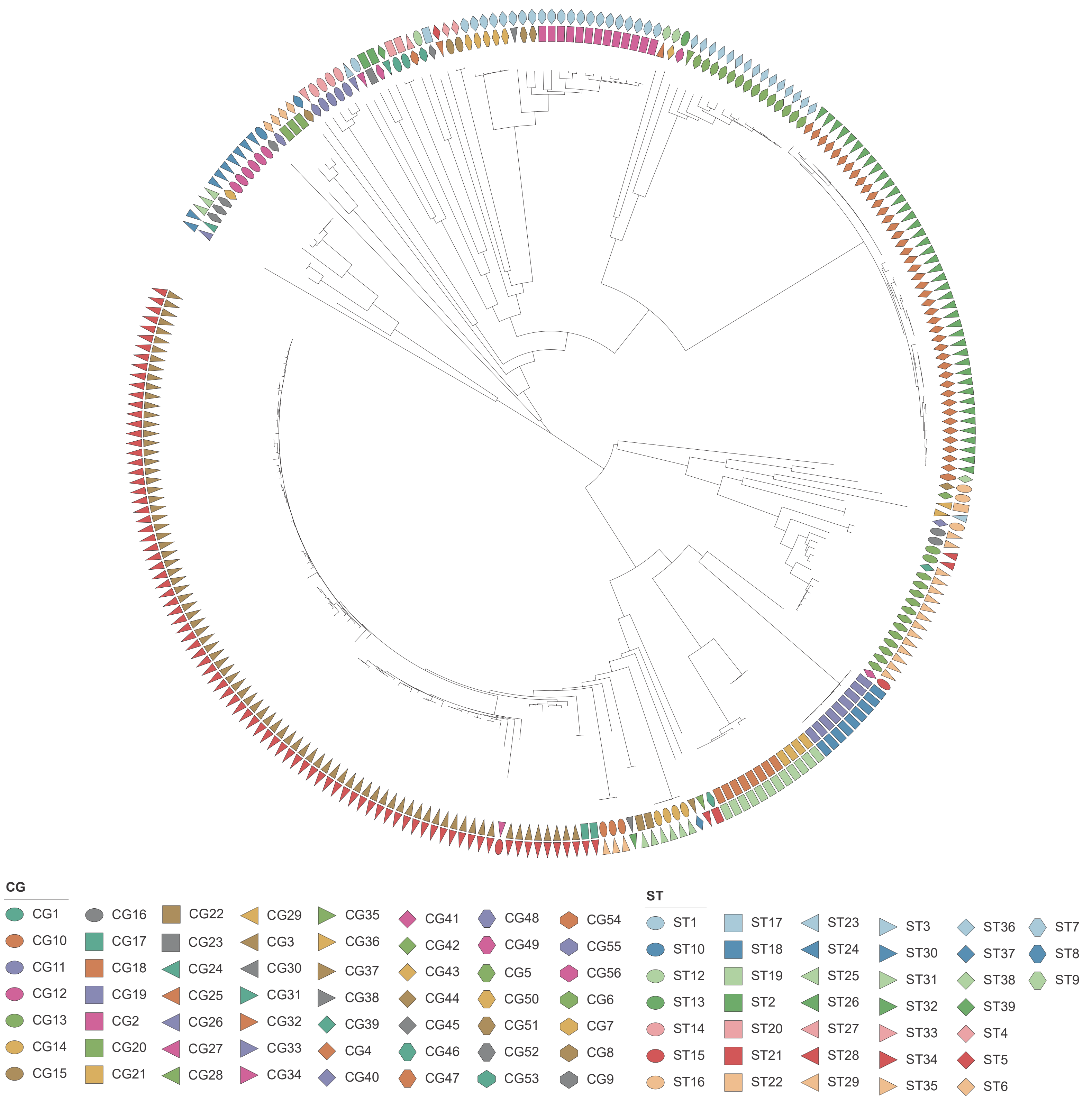
CG
CG1
CG10
CG11
CG12
CG13
CG14
CG15
CG16
CG17
CG18
CG19
CG2
CG20
CG21
CG22
CG23
CG24
CG25
CG26
CG27
CG28
CG29
CG3
CG30
CG31
CG32
CG33
CG34
CG35
CG36
CG37
CG38
CG39
CG4
CG40
CG41
CG42
CG43
CG44
CG45
CG46
CG47
CG48
CG49
CG5
CG50
CG51
CG52
CG53
CG54
CG55
CG56
CG6
CG7
CG8
CG9
ST
ST1
ST10
ST12
ST13
ST14
ST15
ST16
ST17
ST18
ST19
ST2
ST20
ST21
ST22
ST23
ST24
ST25
ST26
ST27
ST28
ST29
ST3
ST30
ST31
ST32
ST33
ST34
ST35
ST36
ST37
ST38
ST39
ST4
ST5
ST6
ST7
ST8
ST9

Figure S3

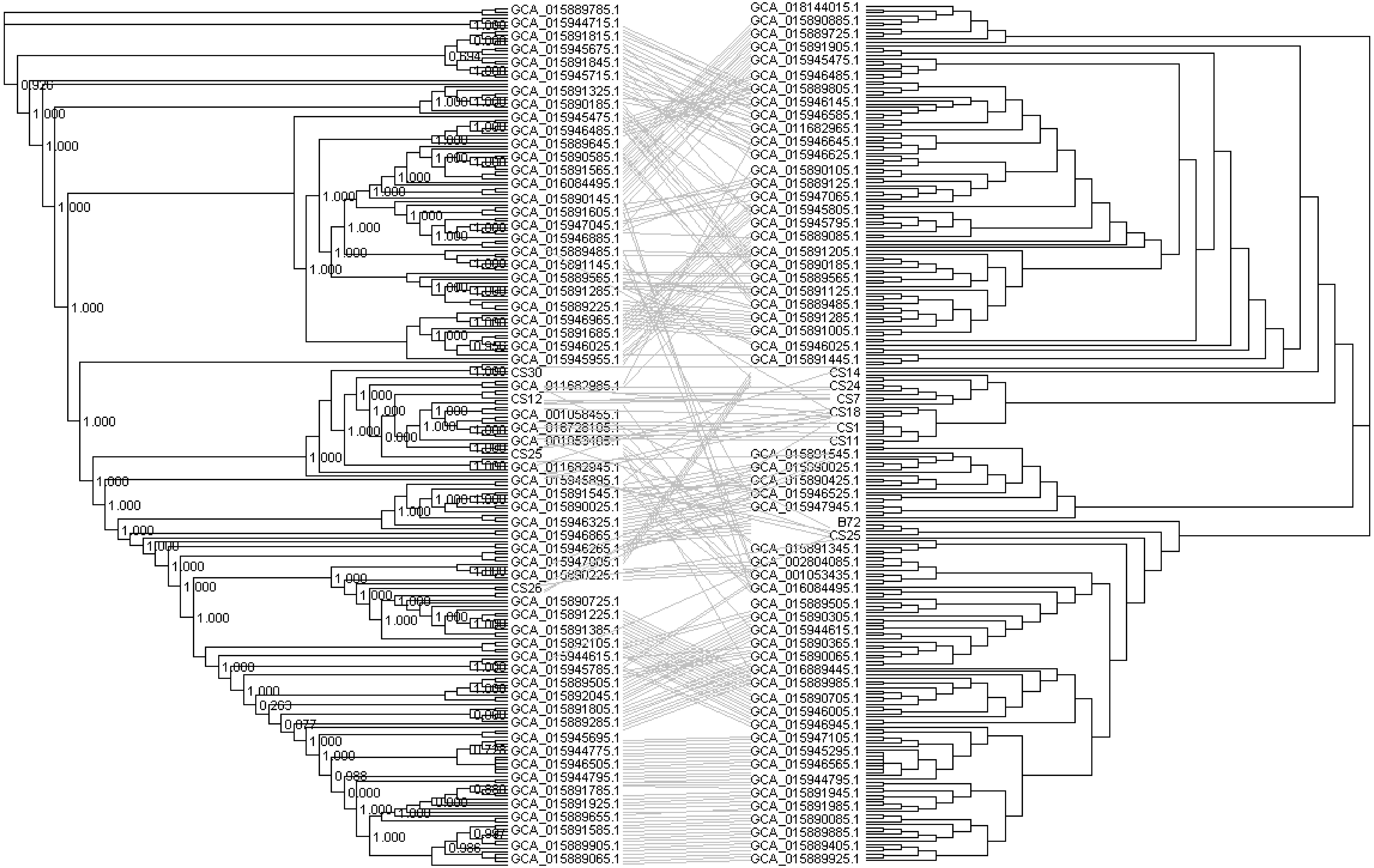
GCA_015889785.1
GCA_015944715.1
GCA_015891815.1
GCA_015945675.1
GCA_015891845.1
GCA_015945715.1
GCA_015891325.1
GCA_015890185.1
GCA_015945475.1
GCA_015946485.1
GCA_015889645.1
GCA_015890585.1
GCA_015891565.1
GCA_016084495.1
GCA_015890145.1
GCA_015891605.1
GCA_015947045.1
GCA_015946885.1
GCA_015889485.1
GCA_015891145.1
GCA_015889565.1
GCA_015891285.1
GCA_015889225.1
GCA_015946965.1
GCA_015891685.1
GCA_015946025.1
GCA_015945955.1
CS30
CS12
CS25
GCA_011682945.1
GCA_015891545.1
GCA_015890025.1
GCA_015946325.1
GCA_015946865.1
GCA_015946265.1
GCA_015947005.1
CS26
GCA_015890725.1
GCA_015891225.1
GCA_015891385.1
GCA_015892105.1
GCA_015944615.1
GCA_015945785.1
GCA_015889505.1
GCA_015892045.1
GCA_015891805.1
GCA_015889285.1
GCA_015945695.1
GCA_015944775.1
GCA_015946505.1
GCA_015944795.1
GCA_015891785.1
GCA_015891925.1
GCA_015889655.1
GCA_015891585.1
GCA_015889905.1
GCA_015889065.1
GCA_018144015.1
GCA_015890885.1
GCA_015889725.1
GCA_015891905.1
GCA_015945475.1
GCA_015946485.1
GCA_015889805.1
GCA_015946145.1
GCA_015946585.1
GCA_011682965.1
GCA_015946645.1
GCA_015946625.1
GCA_015890105.1
GCA_015889125.1
GCA_015947065.1
GCA_015945805.1
GCA_015945795.1
GCA_015889085.1
GCA_015891205.1
GCA_015890185.1
GCA_015889565.1
GCA_015891125.1
GCA_015889485.1
GCA_015891285.1
GCA_015891005.1
GCA_015946025.1
GCA_015891445.1
CS14
CS24
CS7
CS18
CS1
CS11
GCA_015946525.1
GCA_015947945.1
B72
CS25
GCA_002804085.1
GCA_001053435.1
GCA_016084495.1
GCA_015889505.1
GCA_015890305.1
GCA_015944615.1
GCA_015890365.1
GCA_015890065.1
GCA_015889985.1
GCA_015890705.1
GCA_015946005.1
GCA_015946945.1
GCA_015947105.1
GCA_015945295.1
GCA_015946565.1
GCA_015944795.1
GCA_015891945.1
GCA_015891985.1
GCA_015890085.1
GCA_015889885.1
GCA_015889405.1
GCA_015889925.1

Figure S4

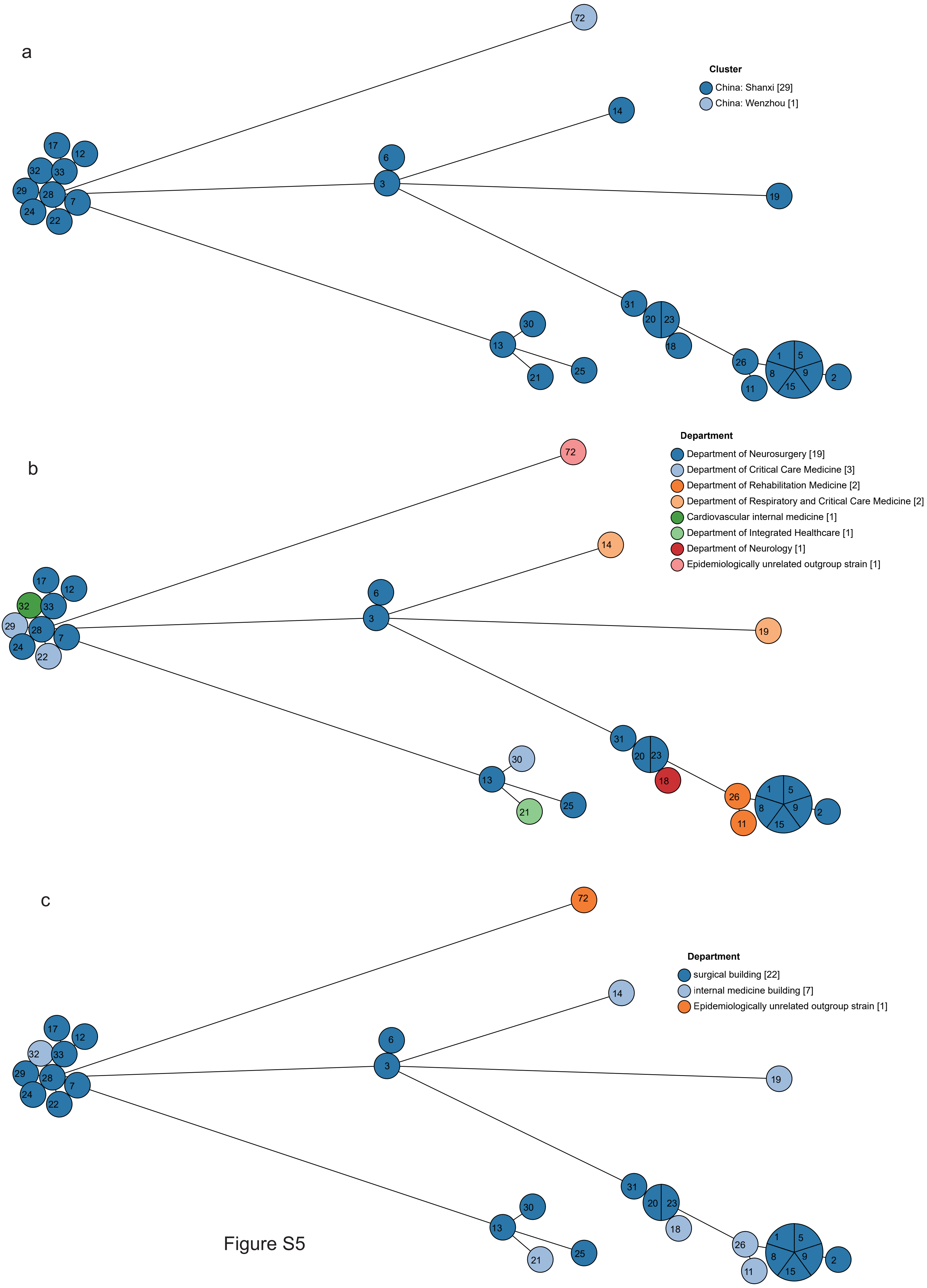
a
Cluster
China: Shanxi [29]
China: Wenzhou [1]
b
Department
Department of Neurosurgery [19]
Department of Critical Care Medicine [3]
Department of Rehabilitation Medicine [2]
Department of Respiratory and Critical Care Medicine [2]
Cardiovascular internal medicine [1]
Department of Integrated Healthcare [1]
Department of Neurology [1]
Epidemiologically unrelated outgroup strain [1]
c
Department
surgical building [22]
internal medicine building [7]
Epidemiologically unrelated outgroup strain [1]

Figure S5

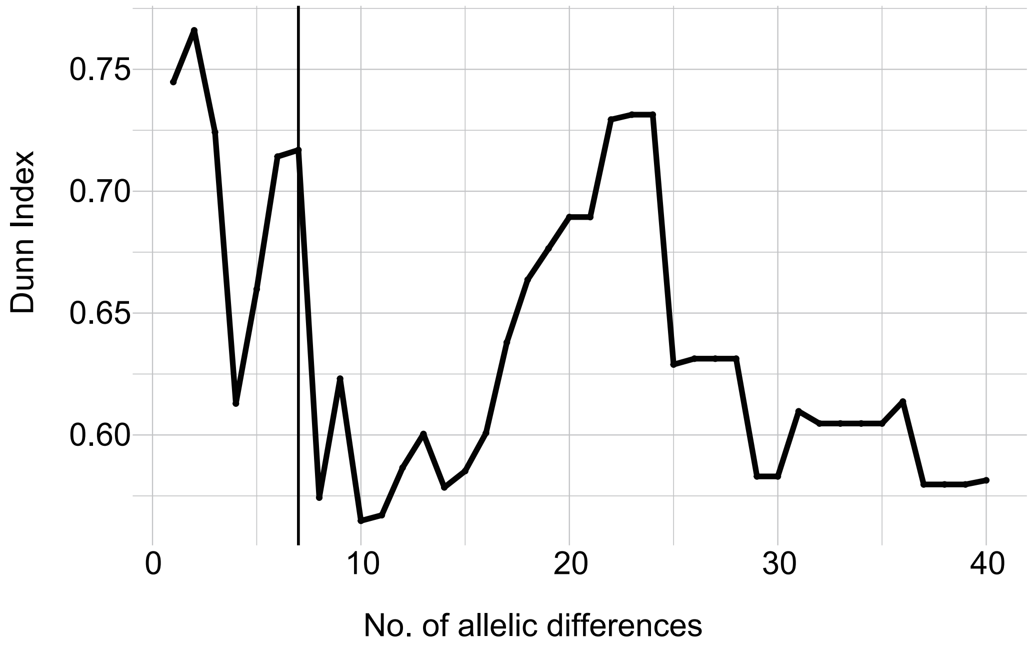
0.75
0.70
0.65
0.60
Dunn Index
0
10
20
30
40
No. of allelic differences

Figure S6
